# A Survey of Brazilian Patients with Oral Lichen Planus Showing No Evidence of Malignancy

**DOI:** 10.1155/2022/5937540

**Published:** 2022-03-16

**Authors:** Dante Migliari, Norberto Sugaya, Silvio Hirota

**Affiliations:** Department of Stomatology, Oral Medicine Division, School of Dentistry, University of São Paulo, São Paulo, Brazil

## Abstract

**Objectives:**

There is conflicting evidence as to whether oral lichen planus (OLP) can undergo malignant transformation into oral squamous cell carcinoma (OSCC). This study aimed to address this issue by analyzing a sample of Brazilian patients with either OLP or OSCC. *Patients and Methods*. This study was conducted in São Paulo, the world's fourth-largest city by population. Two groups of patients were analyzed. The OLP group consisted of 370 patients, while the OSCC group consisted of 154 patients. The OLP patients were followed up for up to 21 years to monitor clinical benefits from the management or changes in the lesion morphology; conversely, patients with OSCC were examined only twice for diagnostic purposes and referred to a specialized center. Data concerning systemic diseases, use of medications, type of oral lesions, and health-risk behaviors were recorded for patients in both groups.

**Results:**

None of the patients with OLP developed OSCC at the lesion site. Only one female patient with erosive OLP developed OSCC in the normal, lesion-free oral mucosa. None of the OSCC patients had concomitant OLP lesions; however, a higher percentage of OSCC cases (17.5%) showed white plaques (most likely oral leukoplakia) as a precursor lesion.

**Conclusion:**

The findings strongly suggest that malignant transformation of OLP is virtually nonexistent in the Brazilian population.

## 1. Introduction

Oral lichen planus (OLP), a chronic, noninfectious inflammatory disease of the oral mucosa, is among the most common types of oral changes observed in clinical settings. The classic OLP is idiopathic in nature; the etiopathogenesis of the disease involves an immune response, mainly by CD8+ lymphocytes, to surface antigens on altered keratinocytes [1].

Epidemiological studies have indicated that OLP affects between 0.1% and 4% of the general population, with a recent meta-analysis reporting a prevalence rate closer to the lower end of the range (approximately 0.9%) [2]. Both middle-aged and older adults are at greater risk for OLP, with a peak incidence between 50 and 59 years of age. The disease occurs less frequently in young people and is quite rare in children. Most studies have also reported that women are on average twice as likely to develop OLP than men, although some studies have reported a female/male ratio of 3 or 4 : 1 [2, 3].

The most controversial issue regarding OLP, which is mainly related to a lack of uniformity among authors on the interpretation of the clinical and histopathological aspects of this disease and, consequently, on its diagnosis, lies in its characterization as a potential oral malignant disease [4]. In this regard, multiple previous studies have reported that OLP exhibits an intrinsic capacity for malignant transformation, i.e., progression to oral squamous cell carcinoma (OSCC) [5–14]. However, many studies reporting the malignant transformation of OLP have been subject to extensive scrutiny, as they failed to comply with strict criteria for OLP diagnosis. Much of the criticism is related to the fact that some lesions diagnosed as OLP, and subsequently undergoing malignant transformation, would be lichenoid dysplasia or that the diagnosis was made based only on the histopathologic findings, which may not be sufficient to provide an accurate characterization of the lesion [15–21]. Additionally, a significant number of studies failed to provide a clear description of OLP cases in which malignant transformation was observed. Furthermore, the reports have described varying rates of malignant transformation of OLP, ranging from 0.4% to 5%.

Nevertheless, there is no study clearly opposing the current trend to characterize OLP as a disease with a malignant potential. A critical review on this issue by Mattsson, Jontell, and Holmstrup [22] highlighted that addressing this controversy requires an analysis of whether the criteria used to diagnose OLP on studies reporting malignant transformation of OLP lesions were thoroughly explicit. On this issue, these authors have found that OLP diagnoses based on well-defined clinical and histopathological criteria were reported by just few selected studies. However, only one study [7] clearly stated that cases showing histopathological signs of dysplasia were excluded. Another concern addressed in the review was that the relevant studies were conducted by experienced oral medicine researchers, which may compromise a judicious assessment on whether the OLP cases that had undergone a malignant transformation did, in fact, involve OLP lesions.

We aimed to address this issue by performing an analysis of two groups of patients examined and diagnosed at our oral medicine clinic: one group composed of patients with OLP and the other consisting of patients with OSCC. Thus, the purpose of this study was to collect clinical evidence to clarify whether OLP should be considered as a potentially malignant disorder in the Brazilian population. These findings hold relevance for the development of suitably reinforcing guidelines regarding the necessity of patients with OLP to return for close follow-ups if the findings reveal a relationship between OLP and OSCC.

## 2. Patients and Methods

### 2.1. OLP Patients

The OLP group consisted of 370 patients (277 females and 93 males; female-to-male ratio: 2.9 : 1) seen consecutively from April 1997 to November 2011 and followed up until 2018 (mean time of follow-up: 7.1 years) at our oral medicine clinic at the Dental School of the University of São Paulo, Brazil. A clinical protocol containing all information regarding oral and medical profiles of the patients was used at the initial consultation and during follow-up examinations. This group included 279 (75.4%) White and 91 (24.6%) non-White patients (mean age, 54.8 years; age range, 25–95 years). OLP was diagnosed according to the criteria described by Kramer et al. [23] and later modified by van der Meij and van der Waal [24]. The clinical findings included the presence of slightly raised white striae (Wickham's striae) and/or white papules usually with bilateral involvement, which are required for the clinical diagnosis of OLP; whereas, the histopathological findings included signs of liquefaction degeneration in the basal cell layer with a well-defined band-like zone of inflammatory infiltrate confined in the superficial part of the connective tissue, which was composed almost exclusively of lymphocytes without epithelial dysplasia. The clinical forms were categorized as reticular, atrophic, erosive, and plaque (plaque-form OLP, specifically requires that reticular striation be present in the lesion periphery and/or in the other areas of the oral mucosa). A conclusive histopathological diagnosis of OLP was obtained in 215 cases (58.1%), while in the remaining 155 cases (41.9%), findings were suggestive of OLP. Patients presenting with lesions lacking some clinical and/or histopathological features of classic OLP were not excluded and were diagnosed as having oral lichenoid lesions (OLLs). A few cases could not be strictly categorized as either OLP or OLL, since they presented only with weak signs of white striation coupled with histopathological findings of nonspecific inflammatory infiltrate. These cases were also included and placed under clinical monitoring with a diagnosis of possible OLL. Patients whose lesions were in direct or close contact with amalgam fillings and later identified as an oral lichenoid contact reaction (OLCR) based on the improvement or resolution of the lesion following amalgam replacement with a nonmetallic material were not included. Additionally, none of the suspected cases of OLP lesions resulted from the use of systemic or topical medication, which is referred to as oral lichenoid drug reaction [25]. All patients with OLP were therefore categorized as having an idiopathic form of the disease. Approximately 13% of these patients missed follow-up. Extraoral lesions of lichen planus (LP) were found in 33 OLP patients (8.9%). Of these, 5 had cutaneous and genital lesions, 20 had only cutaneous lesions, while 8 had only genital lesions. The dermatologic diagnosis of the extraoral lesions was made on a clinical basis, with the exception for lesions located on genital mucosa, whose presumptive LP diagnosis was made on the patients' description of their lesions. Later, all of these patients were referred to the Dermatologic Department of the University of São Paulo for further evaluation and treatment. According to the reports of these patients, no additional biopsies were taken.

A history of systemic disease, along with regular use of medication, was recorded. Exposure to health-related risk factors, such as (but not limited to) cigarette smoking and/or alcohol consumption, was reported in 106 patients (28.6%). Among 71 (25.5%) female patients, 30 (10.8%) smoked cigarettes, 35 (12.6%) consumed alcohol, and 6 (2.1%) had both habits. Among 35 (37.6%) male patients, on the other hand, 13 (14.0%) smoked cigarettes, 15 (16.1%) consumed alcohol, and 7 (7.5%) had both habits. None of them reported being current or former users of illicit drugs.

### 2.2. OSCC Patients

A total of 154 patients with OSCC were examined, underwent biopsies, and received histological diagnoses at our clinic from 2012 to 2019, meaning that approximately 1.6 patients with OSCC were seen at our clinic each month. The main lesions observed at the initial consultation were ulcers (120 cases), followed by white plaques (27 cases) and nodules (7 cases). This group included 45 females and 109 males (female-to-male ratio: 1 : 2.4), and their mean age was 60.4 years (range: 30–97 years). There were 107 White (69.5%) and 47 (30.5%) non-White patients. Cigarette smoking and/or habitual consumption of alcohol were reported in 93 patients (60.4%). Seventeen females (37.7%) either consumed alcohol or smoked, among whom 12 (26.6%) only smoked, while 5 (11.1%) had both habits. Among males, 76 (69.7%) either smoked or consumed alcohol, among whom 34 (31.2%) smoked, 5 (4.6%) only consumed alcohol, and 37 (33.9%) had both habits. Three patients (two males and one female) reported being former consumers of illicit drugs.  Table 1 provides the main findings for both groups. Statistical analysis was performed using the chi-square test with Yates's correction, and the significance was set at *P* < 0.05. This study was approved by the Committee on Ethics of the University of São Paulo (approval reference number: 1.014.994/16).

## 3. Results

The profile of both groups was similar in terms of sex, race, and medical conditions (Table 1). The analysis of cigarette and alcohol use, however, showed an increased exposure to these habits in the OSCC group as compared to that in the OLP group. Statistical significance, however, was only found when the comparison was between males (*P* < 0.05). Females of the OSCC group tended to be more exposed to both habits (smoking and drinking) than those of the OLP group; however, overall, there was no significant difference among females of both groups on these habits. The pattern of consumption was much heavier in the OSCC group, particularly among males. On the other hand, females in the OSCC group were less exposed to cigarette smoking and alcohol consumption as compared to males. In fact, more than half of the females in the OSCC group did not report being exposed to either of these habits in their lives.

Regarding the clinical form and course of OLP, most of OLP patients (52.9%) had the reticular type, while 23.7% had the atrophic form; 13.7% and 9.6% had the erosive and plaque forms, respectively. Mixed forms were also present in some patients, mainly combining reticular and atrophic forms. Patients were monitored on 4-month intervals, on average. For patients experiencing pain, mostly with erosive lesions, they were seen at shorter intervals, until the symptoms had subsided following topical therapy. None of the patients were subjected to a second biopsy during monitoring, unless they had experienced substantial changes on their lesion's morphology, a matter of which was not observed in any of the patients. Over time, in a few cases, changes were observed in the reticular form with lesions presenting attenuated striations. The patients who had experienced the most improvement of their lesions' morphology were those with the erosive form following topical treatment with moderate corticosteroids (betamethasone dipropionate 0.5% in mucosal adhesive formulation) as their lesions turned to a reticular or atrophic form. Around 4.7% had experienced spontaneous resolution of their lesions.

With respect to malignant transformation, none of the patients with OLP developed OSCC in preexisting OLP or OLL lesions. In fact, only one female patient with a histologically confirmed OLP, of erosive form that bilaterally affected the buccal mucosa and lateral border of the tongue, developed OSCC on a clinically normal oral mucosal region on the floor of the mouth after 8 months of follow-up. On the other hand, none of the 154 patients in the OSCC group had concomitant OLP lesions. An important finding was that 27 patients (17.5%) presented with or reported white plaque lesions, most likely oral leukoplakia, at the site of the OSCC lesion. Fifteen of these patients had this lesion on the tongue's dorsal or ventral surfaces, six on the floor of the mouth, three on the gingival mucosa, two on the soft-palate mucosa, and one on the buccal mucosa. Histopathological examinations were available only for 16 patients, with 7 of them showing no signs of dysplasia, while 3 had moderate and 6 had severe dysplasia.

## 4. Discussion

The current trend among clinicians and researchers in the field of oral medicine is to classify OLP as a potentially malignant disease. However, despite the growing number of publications reporting OSCC arising from OLP, some authors are not fully inclined to accept the characterization of OLP as a disease with malignant potential. The lack of full consensus among authors on this issue is primarily based on reports of malignant transformation in OLP cases that did not fulfill the accepted diagnostic criteria and the absence of a detailed morphological description of OLP lesions (mainly in case-series studies) that underwent malignant transformation [26–29]. To highlight this, the study by Robledo et al. [30] involving 1611 patients with OLP, seen over 16 years, did not mention any case with malignant transformation. Aside from the lingering controversy on this matter, some large studies [7, 9–12, 31] have reported OSCCs developed from seemingly true OLP lesions. In these studies, the rate of malignant transformation for OLP varies from 0.4% to 2.5%.

The findings of the present study did not support the characterization of OLP as a disease with malignant potential. Specifically, none of our patients with OLP developed OSCC in preexisting OLP or OLL lesions. In fact, only one female patient presenting with a classic form of OLP, both clinically and histologically, developed OSCC on a clinically normal oral mucosa region on the floor of the mouth after 8 months of follow-up, resulting in an OSCC prevalence of 0.2%. Although this rate is much higher than the reported incidence of OSCC in the Brazilian population (15 per 100000 inhabitants) [32], this case was considered to have occurred at random as it did not arise from a preexisting OLP or OLL lesion. Aside from this study, which covered a period of 21 years (1997–2018), our clinic has recorded no cases of malignant transformation of either OLP or OLL lesions over its 50 years of existence.

Another avenue for investigating the malignant potential of OLP or its risk of OSCC development is to observe the coexistence of OLP lesions in OSCC patients. This approach, which aims to verify the presence of OLP lesions when diagnosing patients with OSCC, is based on the fact that OLP manifests as a multifocal disease in the great majority of cases, thereby, allowing the clinician to observe the presence of an OLP lesion concomitant with OSCC. Moreover, all case-series studies reporting malignant transformation of OLP involved multifocal OLP. In these studies, most of the OSCC cases developed from a lesion that was not initially biopsied. For example, Carbone et al. [12] analyzed 808 OLP cases and observed that only four of the 15 OLP patients who developed OSCC had the carcinoma in the same site of the initial biopsy.

A possible argument opposing this approach (verification of OLP lesions in OSCC patients) would be that OLP patients are normally under periodic monitoring and that the diagnosis of OLP precedes the possible event of malignant transformation. While this may be true, a significant number of OLP cases are diagnosed during routine dental examinations, and furthermore, a high number of patients with asymptomatic OLP lesions could remain undiagnosed for years [30]. Therefore, one possible scenario would involve an oral malignancy arising from a previously undiagnosed multifocal OLP. In such circumstances, the diagnosis of OLP is solely made on a clinical basis, which, per se, is scientifically admissible. This is particularly true for cases in which the clinical manifestations meet the classic characteristics for an OLP diagnosis, characterized by the presence of slightly raised white striae and/or papules with multifocal distribution and bilateral arrangement. Consistent with this, Rödström et al. [31] investigated the association between OSCC and OLP and exclusively resorted to clinical criteria in 38% of 1,028 cases.

Missing follow-up, which occurs in 10–15% of OLP cases, could be another reason for diagnosing OSCC occurring concomitantly with OLP. In this situation, the patient would return for reexamination with a previous diagnosis of OLP or OLL. In another possible scenario, a patient returning for follow-up could be seen by a different clinician in a different clinical setting other than the one where the initial diagnosis of OLP had been made.

Only one study [33] provided data on the coexistence of OLP in OSCC diagnosis. These authors reported that out of 323 patients with OSCC, 71 (22%) had either OLP or OLL as a precursor lesion. This indicates a very high percentage of OLP lesions associated with malignant transformation, much higher than that associated with oral leukoplakia. Therefore, this finding is not representative of the actual patient population. One of the highest quality studies on this issue [7] reported a rate of malignant transformation of OLP at 1.5%.

Our investigation also aimed to verify the presence of OLP lesions in 154 patients with OSCC and did not show any case of OSCC that could have resulted from a preexisting OLP lesion. As stated previously, OLP manifests most frequently as a multifocal oral mucosal lesion. Therefore, concomitant OLP and OSCC lesions would theoretically be easily recognizable on examination. In this study, oral leukoplakia occurred as a precursor lesion in 27 (17.5%) patients with OSCC. During the oral examination, these patients had reported the presence of long-term white plaques at the site of the development of OSCC or showed a white plaque in conjunction with malignancy.

In order to validate the reliability of the findings reported in this study, we would like to highlight that our clinic is affiliated with the University of São Paulo, the country's most renowned institution for teaching and research at the undergraduate and graduate levels. Apart from being a public institution, the University of São Paulo—including medical and dental schools and diverse clinical specialties—serves the population of São Paulo, especially providing services for its predominantly low-income citizens. As a result, our oral medicine clinic is one of the main centers for referrals of patients experiencing oral mucosal diseases.

Under this setting and with clinical experience that spanned over four decades, the most common oral mucosal disease showing a malignant potential at our clinic was oral leukoplakia. While a few cases of oral erythroplakia with malignant transformation have been recorded, no case of OLP or OLL progressing to malignancy has been reported to date. These findings indicate that the malignant potential of OLP is virtually nonexistent in the Brazilian population.

In contrast with most of studies focusing on malignant transformation of OLP, the present survey did not support classifying OLP as a disease with a malignant potential. There were some studies [7, 12] showing, apparently convincingly, that patients with OLP had an increased risk for OSCC development; while, the findings of another study in a Swedish population [28] did not characterize OLP as disease that would increase the individuals' predisposition for OSCC development as compared with that of general population.

It seems that conflicting data on this issue will remain, despite all attempts to find a common ground among authors. In an attempt to address this matter, we believe that researchers tasked with peer reviewing future studies on this issue should be more critical of statements assuring that all patients have met the criteria for the diagnosis of OLP, as this is usually unattainable. Moreover, for future studies aiming to elucidate the true potential for malignant transformation in patients with OLP, it is essential that such studies include only cases of preexisting, clinically and histologically proven OLP, and a detailed morphological description of OLP lesions that underwent malignant transformation.

## Figures and Tables

**Table 1:** Clinical profile and risk habits (smoking and alcohol consumption) in patients with oral lichen planus (OLP) and oral squamous cell carcinoma (OSCC).

	Patients					
	OLP (*n* = 370)			OSCC (*n* = 154)		
	Male (%)	Female (%)	Total (%)	Male (%)	Female (%)	Total (%)
Number	93 (25.1)	277 (74.8)	370 (100)	109 (70.8)	45 (29.2)	154 (100)
Mean age (range) (years)	53.2 (36–74)	54.1 (25–95)	54.8 (25–95)	58.5 (30–97)	66.1 (39–91)	60. 4 (30–97)

Race						
White	67 (72.0)	212 (76.4)	279 (75.4)	74 (67.9)	33 (73.3)	107 (69.5)
Non-White	26 (28.0)	65 (23.5)	91 (24.6)	35 (32.1)	12 (26.6)	47 (30.5)

History of disease						
Hypertension	22 (23.6)	79 (28.5)	101 (27.3)	28 (25.6)	13 (28.8)	41 (26.6)
Diabetes	20 (21.5)	48 (17.3)	68 (18.3)	16 (14.6)	6 (13.3)	22 (14.2)
Hypothyroidism	1 (1.0)	32 (11.5)	33 (8.9)	–	4 (8.8)	4 (2.5)
Hepatitis B	6 (6.4)	9 (3.2)	15 (4.0)	4 (3.6)	1 (2.2)	5 (3.2)
Hepatitis C	2 (2.1)	5 (1.8)	7 (1.8)	1 (0.9)	2 (4.4)	2 (1.9)
Anemia history	3 (3.2)	36 (13.0)	39 (10.5)	6 (5.5)	5 (11.1)	11 (7.1)
Allergy	5 (5.3)	41 (14.8)	46 (12.4)	7 (6.4)	6 (13.3)	13 (8.4)
Autoimmune disease	–	5 (1.8)	5 (1.3)	–	2 (4.4)	2 (1.2)
Use of immunosuppressor medication	–	3 (1.0)	3 (0.8)	–	2 (4.4)	2 (1.2)

Habits						
No habits	58 (62.3)	206 (74.4)	264 (71.3)	33 (30.3)	28 (62.2)	61 (39.6)
Either smoking or drinking	35 (37.6)^*∗∗*^	71 (25.6)^**†**^	106 (28.6)^*∗*^	76 (69.7)^*∗∗*^	17 (37.7)^**†**^	93 (60.4)^*∗*^
Smokers	13 (14.0)	30 (10.8)	43 (11.6)	34 (31.2)	12 (26.6)	46 (29.9)
Drinkers	15 (16.1)	35 (12.6)	50 (13.5)	5 (4.6)	–	5 (3.2)
Smoker and drinker	7 (7.5)	6 (2.1)	13 (3.5)	37 (33.9)	5 (11.1)	42 (27.3)

^*∗*^Significant *P* value = 0.001. ^*∗∗*^Significant *P* value = 0.0001. ^**†**^Nonsignificant *P* value = 0.12.

## Data Availability

The data used to support the findings of the study are available from the corresponding author upon reasonable request.
